# Modulation of hippocampal theta and hippocampal‐prefrontal cortex function by a schizophrenia risk gene

**DOI:** 10.1002/hbm.22778

**Published:** 2015-03-10

**Authors:** Helena Cousijn, Elizabeth M. Tunbridge, Michal Rolinski, George Wallis, Giles L. Colclough, Mark W. Woolrich, Anna C. Nobre, Paul J. Harrison

**Affiliations:** ^1^ Department of Psychiatry University of Oxford Warneford Hospital Oxford, United Kingdom; ^2^ Oxford Centre for Human Brain Activity University of Oxford Warneford Hospital Oxford United Kingdom; ^3^ Nuffield Department of Clinical Neurosciences University of Oxford John Radcliffe Hospital Oxford United Kingdom

**Keywords:** hippocampus, magnetoencephalography, oscillation, psychosis, functional magnetic resonance imaging, *ZNF804A*

## Abstract

Hippocampal theta‐band oscillations are thought to facilitate the co‐ordination of brain activity across distributed networks, including between the hippocampus and prefrontal cortex (PFC). Impairments in hippocampus‐PFC functional connectivity are implicated in schizophrenia and are associated with a polymorphism within the *ZNF804A* gene that shows a genome‐wide significant association with schizophrenia. However, the mechanisms by which *ZNF804A* affects hippocampus‐PFC connectivity are unknown. We used a multimodal imaging approach to investigate the impact of the *ZNF804A* polymorphism on hippocampal theta and hippocampal network coactivity. Healthy volunteers homozygous for the *ZNF804A* rs1344706 (A[risk]/C[nonrisk]) polymorphism were imaged at rest using both magnetoencephalography (MEG) and functional magnetic resonance imaging (fMRI). A dual‐regression approach was used to investigate coactivations between the hippocampal network and other brain regions for both modalities, focusing on the theta band in the case of MEG. We found a significant decrease in intrahippocampal theta (using MEG) and greater coactivation of the superior frontal gyrus with the hippocampal network (using fMRI) in risk versus nonrisk homozygotes. Furthermore, these measures showed a significant negative correlation. Our demonstration of an inverse relationship between hippocampal theta and hippocampus‐PFC coactivation supports a role for hippocampal theta in coordinating hippocampal‐prefrontal activity. The *ZNF804A*‐related differences that we find in hippocampus‐PFC coactivation are consistent with previously reported associations with functional connectivity and with these changes lying downstream of altered hippocampal theta. Changes in hippocampal‐PFC co‐ordination, driven by differences in oscillatory activity, may be one mechanism by which *ZNF804A* impacts on brain function and risk for psychosis. *Hum Brain Mapp 36:2387–2395, 2015*. © **2015 The Authors Human Brain Mapping Published by Wiley Periodicals, Inc.**

## INTRODUCTION

Oscillations regulate the levels of neuronal excitability within the brain to support efficient information processing [Buzsaki and Draguhn, 2004]. Theta‐band oscillatory activity (∼4–8 Hz in humans) is generated in the hippocampal region [White et al., 2000] and is synchronized across distributed neuronal networks [Buzsaki and Draguhn, 2004]. As well as playing an important role in spatial navigation, theta oscillations are believed to co‐ordinate the activity of the hippocampus and prefrontal cortex (PFC) to mediate effective cognitive function [Colgin, 2011; Fujisawa and Buzsaki, 2011]. As one example, theta oscillations, acting in concert with the cortical gamma band (30–100 Hz) activity that is also strongly implicated in cognitive function [Uhlhaas et al., 2008], are proposed to provide a neural coding scheme to enable items to be held in working memory in a temporally organized manner [Lisman and Buzsaki, 2008]. Consistent with this proposal, theta power measured over frontal regions increases during PFC‐dependent working memory task performance and scales with task difficulty [Gevins et al., 1997; Gundel and Wilson, 1992].

Disrupted hippocampus‐PFC interactions have long been implicated in schizophrenia [Weinberger et al., 1992]. More recently, it has been suggested that abnormalities in the functional coupling of these regions may be a common “weak link” across multiple psychiatric disorders [Godsil et al., 2013]. Patients with schizophrenia also show abnormalities in oscillatory activity, notably within the theta, and gamma bands [Uhlhaas et al., 2008]. It is also not clear whether genetic factors associated with schizophrenia risk contribute to these changes, although this is an attractive hypothesis given that brain oscillations are highly heritable [van Beijsterveldt et al., 1996]. It is not known whether the differences in oscillatory activity seen in patients relate to changes in hippocampal‐PFC co‐ordination. However, rodent models related to schizophrenia support this hypothesis and suggest that these changes can be seen in subjects at rest, as well as during the task‐engaged state [Dickerson et al., 2012], in line with a surge in interest of the utility of studying task‐independent network activity to examine healthy brain function and to address a range of clinical questions [Satterthwaite and Baker, 2015; van Diessen et al., 2014]. Indeed, it has been recently proposed that schizophrenia might result from disorder in the spatiotemporal patterns of activity in the brain's resting state [Northoff, 2015].

Genome‐wide association studies support an association between the rs1344706 (A[risk]/C[nonrisk]) polymorphism in the *ZNF804A* gene and psychosis, including schizophrenia [Schizophrenia Working Group of the Psychiatric Genetics Consortium, 2014; Williams et al., 2011]. Little is known about the function of *ZNF804A*, although it is predicted to be a transcription factor, and is expressed in human brain throughout the lifespan [Hill and Bray, 2012; Tao et al., [Link]]. The risk polymorphism is associated with altered hippocampus‐PFC functional connectivity assessed using seed‐based connectivity measures [Esslinger et al., 2011; Paulus et al., 2013; Rasetti et al., 2011], although there are some inconsistencies and complexities to this relationship, discussed further below. However, the mechanisms by which genetic variation in *ZNF804A* impacts on hippocampus‐PFC functional connectivity are unknown. Therefore, we investigated the impact of *ZNF804A* genotype on hippocampal and PFC coactivity and its relationship with hippocampal theta, using a multimodal imaging approach in healthy young adult volunteers in the absence of a cognitive task (referred to subsequently as the “resting state,” for brevity). Specifically, we studied regions showing coactivity with the hippocampal network [Smith et al., 2009] using both functional magnetic resonance imaging (fMRI) and magnetoencephalography (MEG), focussing on the theta band. We focussed on the hippocampal network, given the key role of this region in generating theta‐band activity, and because activity in this region emerges as a discrete and clearly defined network from independent components analyses (ICAs) of both MEG and fMRI data [Luckhoo et al., 2012; Smith et al., 2009]. This experimental design also allowed us to investigate whether there is a relationship between hippocampal theta and cortical, particularly PFC, function, as would be predicted if hippocampal theta is relevant for co‐ordinating long‐range brain activity.

## MATERIALS AND METHODS

### Participants

The study was approved by the NHS South Central, Berkshire Research Ethics Committee: 11/SC/0053. Healthy, right‐handed participants aged 18–35 were recruited via advertisement. Exclusion criteria included a history of psychiatric or neurological illness, the use of any medication which might affect brain function and the presence of any metal in or on the body that could not be removed. Participants who fulfilled the inclusion criteria were genotyped for *ZNF804A* rs1344706 using the appropriate Taqman® SNP Genotyping Assay (Applied Biosystems, Carlsbad, CA). Only homozygotes were selected for participation in the final study. All participants denied current use of illicit drugs. Details of the final sample are included in the “Results” section, below.

### Neuroimaging

Magnetic resonance imaging (MRI) data were acquired at the University of Oxford's Centre for Clinical Magnetic Resonance Imaging centre, using a 3T Siemens TIM Trio scanner (Siemens AG, Erlangen, Germany) with a 32‐channel head coil. Functional imaging consists of 34 T2*‐weighted echo‐planar image axial oblique slices that began at the cerebral vertex and encompassed the cerebrum and the majority of the cerebellum. One hundred and eighty volumes were acquired per subject, giving a total scan time of 6 min (repetition time (TR) = 2 s; echo time (TE) = 28 ms; flip angle = 89°; field of view (FOV) = 192 × 192; matrix size = 64 × 64). Participants were asked to keep their eyes open and fixate on a white cross in the center of a gray screen. A high‐resolution three‐dimensional (3D) T1‐weighted structural scan was also acquired, using the following parameters: 224 slices (0.8‐mm thick); distance factor = 50%; FOV (read) = 256 mm; FOV (phase) = 68.8%; matrix = 174 × 192; TR = 3 s; TE = 4.8 ms; TI = 1100 ms; flip angle = 8°; 1 concatenation; bandwidth = 220 Hz; echo spacing = 9.6 ms.

MEG data were acquired at the Oxford Centre for Human Brain Activity, using the Elekta NeuroMag MEG System (Elekta, Stockholm, Sweden). Participants were seated in the MEG scanner and asked to keep their eyes open and to fixate on a white cross in the center of a gray screen. Resting state activity was measured for 6 min. The signal was digitized at a sampling rate of 1 kHz, with a high‐pass filter of 0.03 Hz and a low‐pass filter of 330 Hz. A magnetic digitizer (FastTrak 3D; Polhemus, Colchester, VT) was used to measure the relative positions of four head‐position indicator coils and three anatomical landmarks (nasion, left, and right auricular points), which were used for coregistration of the sensor montage to the individual's structural MRI scan.

### fMRI Analysis

Data were analyzed using the Oxford Centre for Functional Magnetic Resonance Imaging of the Brain's Software Library (FSL; version 4.1.10) tools (http://www.fmrib.ox.ac.uk/fsl). Individual preprocessing consisted of motion correction, brain extraction, spatial smoothing using a Gaussian kernel of full‐width at half‐maximum of 6 mm, and high‐pass temporal filtering. FSL's Multivariate Exploratory Linear Decomposition into Independent Components (version 3.10) was used to perform probabilistic ICA on individual datasets, which were then denoised using FMRIB's ICA‐based Xnoiseifier v1.05 beta [Salimi‐Khorshidi et al., 2014]. Individual fMRI volumes were first registered to the individual's anatomical scan using FMRIB's linear image registration tool followed by a boundary‐based registration approach. Registration from anatomical to standard (Montreal Neurological Institute; MNI) space was conducted using FMRIB's nonlinear image registration tool. Gray matter maps were obtained using FSL‐Voxel‐basedMorphometry (VBM) (version 1.1) with default settings [Douaud et al., 2007].

A standard set of resting‐state networks (RSNs), obtained in a separate group of healthy participants [Smith et al., 2009], was used for both fMRI and MEG analyses. The dual regression method was used to test the effects of *ZNF804A* genotype on hippocampal network coactivation [Filippini et al., 2009], with gray matter maps as voxel‐wise covariates. This yielded statistical maps of parameter estimates (PEs) for individual participants, describing the extent of every voxel's involvement in the hippocampal network. These individual maps were incorporated into a single‐4D file for each group, and genotype differences were assessed using voxel‐wise, nonparametric, permutation‐based testing (5,000 permutations), using FSL's Randomise (version 2.1), and a family‐wise error threshold‐free cluster estimation (TFCE) significance level of *P* < 0.05. A one‐sample *T*‐test (also implemented in randomize) was used to identify voxels showing significant coactivation with the hippocampal network in the whole group.

To visualize the results and to permit correlations between fMRI and MEG data, individual PE values were extracted from each individual's custom hippocampal map, using the significant voxels within the prefrontal cluster (Table 1) as a binary mask. PE values were non‐normally distributed and so were compared between genotype groups using a Mann–Whitney *U* test, implemented in SPSS Statistics 20 (IBM, Armonk, NY).

**Table 1:** Regions showing significantly greater coactivation with hippocampal network in risk versus nonrisk homozygotes determined using fMRI (*P* < 0.05 FWE corrected; cluster ≥ 10 voxels)

Cluster location	Cluster size	Most significant voxel (MNI co‐ordinates: *x*, *y*, *z*)
L superior frontal gyrus	13	−24, 28, 48
R middle temporal gyrus	10	52, −32, −6
L calcarine cortex	40	−18, −76, 4
R calcarine cortex	36	14, −70, 10
R calcarine cortex	14	2, −70, 10

### MEG Image Analysis

Data analysis used Matlab scripts that incorporate tools within FSL [Smith et al., 2004] and the Matlab toolboxes SPM8 (http://www.fil.ion.ucl.ac.uk/spm/) and FieldTrip [Oostenveld et al., 2011]. Data from both the 204 gradiometers and 102 magnetometers were included in the analysis. Individual datasets were preprocessed as follows. First, noisy channels were manually identified and removed, before applying MaxFilter™ v2.2 (Elekta), with signal space separation and movement correction. Data were downsampled to 250 Hz, and epochs containing artefacts associated with body movement or external sources were marked manually. Periods with artefacts were kept in the data but excluded at critical points in the analysis where they would introduce errors (e.g., the estimation of the covariance matrix in the beamformer). A semiautomatic ICA‐based artefact rejection method that identifies noise components associated with line noise (50 Hz), eye blinks, and heartbeat was then applied. All ICA components were inspected, and those judged to represent noise (based on their spatial topography, timecourse, and frequency spectrum) were removed from both the data and the lead fields. Finally, a high‐pass (1 Hz) filter was applied, and the data were inspected again to allow removal of any remaining epochs containing artefacts. Preprocessed data were coregistered to standard sensor space based on the individual's head position information and structural scan, using SPM8. An overlapping local spheres forward model [Huang et al., 1999], implemented in FieldTrip, was used to define the head shape of each subject and to calculate lead fields to model the MEG signal at each grid point. A linearly constrained minimum variance (LCMV) beamformer was then applied [Woolrich et al., 2011] to estimate the sources of theta‐band signals. As these data had been MaxFiltered (this reduces the dimensionality of the data to ∼64) and also consisted of multiple sensor types, two additional steps were necessary: (1) to make data across sensor types compatible, each sensor type was normalized by its smallest eigenvalue (restricted to the first 60 eigenvectors); (2) When inverting the data covariance matrix, the rank of the data was fixed to be 60 [Woolrich et al., 2011]. Data bandpass filtered to the theta band (4–8Hz) was projected into source space. The 4–8 Hz band was used as this is the commonly found range for theta in human studies and is the optimal theta range for RSN analyses [Axmacher et al., 2010; Lega et al., 2012; Luckhoo et al., 2012]. A timecourse of theta oscillatory activity was calculated for each grid point, which was 6 mm apart (and is hereafter referred to as 6 mm^3^ voxels). A Hilbert transform was then applied to each voxel to derive the amplitude of oscillatory activity. The Hilbert envelope was then downsampled temporally, by dividing each envelope timecourse into 2 s nonoverlapping windows and averaging the data within these windows. This has consistently been shown to be a robust way of detecting stationary functional connectivity [Brookes et al., 2011a, 2011b; Luckhoo et al., 2012]. Both beamformer‐weights‐normalized and nonbeamformer‐weights‐normalized envelopes were estimated (these will be used later in the group‐level analysis and see [Luckhoo et al., 2014] for a description of the beamformer weights).

We used ICA (implemented using FastICA) [Hyvarinen, 1999] to decompose the envelopes concatenated across subjects into 25 temporally independent components. The spatial maps of these components (or networks) indicate brain areas that contain correlated activity over time with each other [Brookes et al., 2011b]. Resulting networks were examined to assess whether hippocampal networks similar to those observed in fMRI analyses can be seen in MEG data. However, to allow a consistent analysis approach to be used for both MEG and fMRI data, a well‐validated, standard set of RSN ICA maps obtained using fMRI [Smith et al., 2009] was used for the main analysis.

A MEG‐adapted dual‐regression (DR_MEG_) approach analogous to that used for fMRI data was used to compare hippocampal network theta between the two *ZNF804A* genotype groups. In the first stage of DR_MEG_, we performed a spatial regression of the standard group‐level ICA maps, using the well‐validated standard RSN ICA maps detailed above [Smith et al., 2009], on the weights‐normalized envelopes over data concatenated over all participants to yield the set of independent component time courses. In the second stage, time courses were separated into subject‐specific blocks. For each subject, we performed a temporal regression of the component time course segment from the nonweights‐normalized downsampled envelopes. This gave a spatial map for each RSN that is specific to each subject but critically has an unbiased estimate of the true variance of activity for that RSN, which is essential for all subsequent multisubject statistics [Luckhoo et al., 2014].

These individual subject maps were incorporated into a single 4D file for each group, and genotype differences were assessed using voxel‐wise, nonparametric, permutation‐based testing (5,000 permutations), using FSL's Randomise (version 2.1), and a family‐wise error TFCE significance level of *P* < 0.05. In addition to the between‐groups comparison, we also calculated a single‐group average to identify regions showing significant coactivation with the hippocampal network across all subjects over all groups.

To permit correlations between fMRI and MEG data, we calculated a binary mask by extracting PEs from the individual hippocampal spatial maps (using the spatial maps from the dual regression, i.e., the maps that were also concatenated into a 4D file and used to find genotype differences) and thresholding them to include only those voxels that showed significant genotype group differences.

### Correlations Between fMRI and MEG measures

For both fMRI and MEG data, PEs were extracted from subject‐specific spatial hippocampal maps using the cluster of interest showing a significant genotype group difference (PFC for fMRI data; right hippocampus for MEG data) as a binary mask, as described above. Correlations between fMRI and MEG data were explored using Spearman's correlation, implemented in SPSS Statistics 20.

## RESULTS

The sample consisted of 25 AA “risk” homozygotes (11 male/14 female; age [mean ± SEM] = 23.4 ± 0.72 years) and 25 CC “nonrisk” homozygotes (12 male/13 female; age = 24.6 ± 0.88 years). The homozygote groups were well matched for sex (Chi‐square test: *χ* ^2^ = 0.08; d.f. = 1; *P* = 0.777) and age (*t*‐test: *t* = −0.982; d.f. = 48; *P* = 0.331). Consistent with recent findings in a large sample of volunteers [Cousijn et al., 2012], there were no genotype group differences in gray matter maps (*P*'s > 0.32 after FWE correction for multiple comparisons).

### Hippocampal Network Activity Determined in fMRI Signal and MEG Theta‐Band Activity at Rest

The whole‐group ICA of MEG theta‐band activity successfully identified left‐ and right‐lateralized hippocampal networks (Supporting Information Fig. 1A), confirming that hippocampal theta‐band network activity can be observed in the resting state. These networks were similar to the (bilateral) hippocampal network previously identified using fMRI (Supporting Information Fig. 1B) and used for subsequent stages of the analysis.

**Figure 1:**
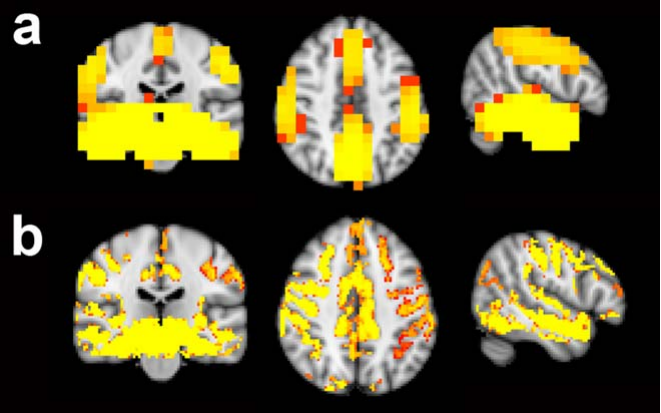
Hippocampal network co‐activation is consistent across fMRI and MEG theta‐band activity. Regions showing co‐activation with the hippocampal network in the whole group of participants, determined (a) in MEG theta‐band activity and (b) using fMRI. Images show regions coactivated with the hippocampal network at a threshold of *P* < 0.05 (after FWE correction for multiple comparisons).

We next examined regions showing significant coactivity with the hippocampal network in the whole group of participants, by comparing the TFCE‐corrected statistical maps of whole‐group hippocampal network coactivations derived (as described above) for fMRI and MEG. The patterns of coactivation observed using fMRI and MEG (focussing on the theta‐band) were compared using FSL cross correlation and were found to be highly correlated (Fig. 1; *r* = 0.54; *P* < 0.000006) estimated using Fisher's r‐to‐*z* transform and a conservative estimate of spatial d.f. = 60 based on the number of independent resolution elements, as described previously [Smith et al., 2009]. Regions showing coactivity with the hippocampal network included, bilaterally, the amygdala and frontal, temporal, and precuneus cortices. Thus, widespread coactivations, that were similar between fMRI and MEG theta‐band activity, could be detected in participants at rest.

### *ZNF804A* Modulates Hippocampal‐PFC Coactivation

**Figure 2:**
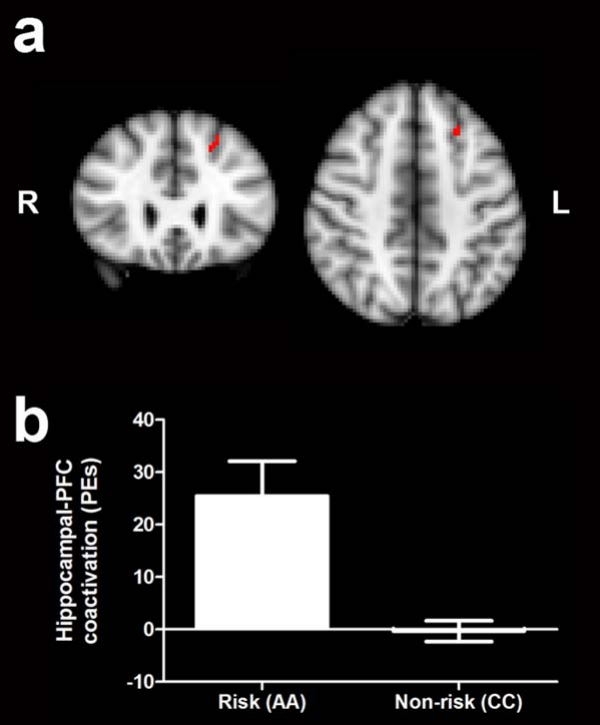
Hippocampal‐PFC coactivation is modulated by *ZNF804A* genotype. (a) Coactivation of the hippocampal network and superior frontal gyrus is greater in *ZNF804A* risk versus nonrisk homozygotes (*P* < 0.05 after FWE correction for multiple comparisons; *n* = 25 per group). (b) Risk homozygotes show positive hippocampal‐PFC coactivation which is absent in nonrisk homozygotes (Mann–Whitney *U*: *P* = 0.00007; *n* = 25 per group).

We used fMRI to investigate brain regions that showed significant correlations in activity with the hippocampal network. We found greater correlation between PFC and hippocampal network activity in risk versus nonrisk homozygotes (Fig. 2A; Table 1), consistent with previous findings linking the risk variant with greater hippocampal‐PFC functional connectivity than the nonrisk allele [Esslinger et al., 2009; Paulus et al., 2013]. More specifically, risk homozygotes showed a coactivation of the left superior frontal gyrus and hippocampal network that was absent in nonrisk homozygotes (Mann–Whitney *U* test: *P* = 0.000007; Fig. 2B). Additionally, hippocampal coactivation with the bilateral calcarine cortex and right middle and superior temporal gyri (Table 1) was greater in risk versus nonrisk homozygotes. There were no regions showing greater hippocampal network coactivation in nonrisk versus risk homozygotes.

### *ZNF804A* Modulates Intrahippocampal Theta

**Figure 3:**
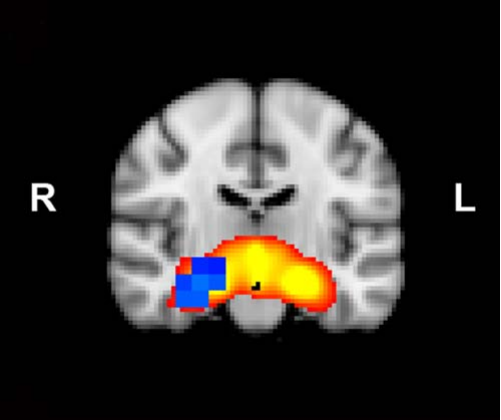
Hippocampal theta is modulated by *ZNF804A* genotype. Risk allele homozygotes show decreased coactivation of the right hippocampus (shown in blue; *P* < 0.05 after FWE correction for multiple comparisons; *n* = 25 per group) with the rest of the hippocampal network (red heat map thresholded at 3 > *Z* > 8), versus nonrisk homozygotes.

We used MEG to examine regions showing activity correlated with that of the hippocampal network [Smith et al., 2009], focussing on the theta band. Using dual regression, we found greater correlated activation of the right hippocampus with the rest of the hippocampal network in nonrisk versus risk homozygotes (Fig. 3). This difference was limited to low‐frequency bands (specifically, theta and alpha [8–12 Hz]; Supporting Information Fig. 2). No other regions showed changes in hippocampal network theta in nonrisk versus risk homozygotes, nor were there any regions showing significant differences for the risk versus nonrisk contrast. There were no differences in MEG power in the entire hippocampal network between groups (Supporting Information Fig. 3).

### Intrahippocampal Theta Correlates with Hippocampal‐PFC Coactivity

**Figure 4:**
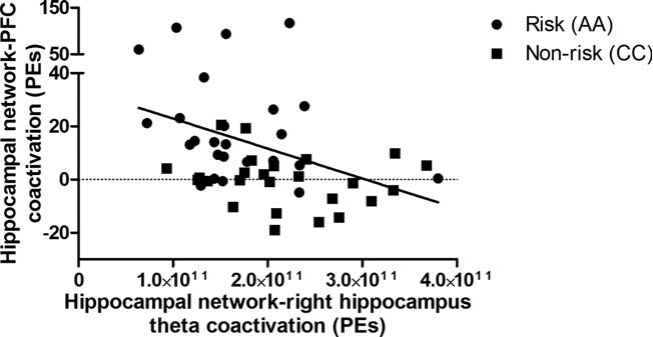
Intrahippocampal theta (determined using MEG; see Fig. 2) and hippocampal‐PFC coactivation (determined using fMRI; see Fig. 1) are inversely related (Spearman's rho = −0.40; *P* = 0.005; *n* = 25 per group; see Supporting Information). Correlations are negative within both groups (Risk [circles]: Spearman's rho = −0.23; nonrisk [squares]: Spearman's rho = −0.21).

We performed a correlational analysis to investigate whether there is a relationship between the hippocampal‐PFC coactivation and intrahippocampal theta measures that showed *ZNF804A* genotype‐related differences. The presence of such a relationship would be consistent with *ZNF804A*‐related differences in hippocampal‐PFC co‐ordination lying downstream of an alteration in hippocampal theta, as would be predicted from the literature. In line with this proposal, right hippocampal theta (from MEG) and hippocampal‐PFC coactivation (from fMRI) were inversely correlated (Spearman's rho = −0.40; *P* = 0.005). Correlations were negative within both groups (Risk homozygote Spearman's rho = −0.23 [*P* = 0.27]; nonrisk homozygotes Spearman's rho = −0.21 [*P* = 0.31]) and do not differ from one another (Fisher's *r* to *z* transformation [http://vassarstats.net/rdiff.html]: *z* = −0.05; *P* = 0.944 [Myers and Sirois, 2006]), indicating that this inverse relationship is present in both risk and nonrisk carriers and therefore consistent with a general relationship between hippocampal theta and hippocampal‐PFC coactivation (Fig. 4).

## DISCUSSION

As outlined above, hippocampal theta is thought to play an important role in co‐ordinating hippocampal‐PFC activity. This circuitry is dysfunctional in patients with schizophrenia, and a polymorphism in *ZNF804A* associated with risk for schizophrenia is linked with altered hippocampus‐PFC functional connectivity. The aim of this study was two‐fold: to investigate whether *ZNF804A* is associated with hippocampal theta and hippocampal‐PFC coactivation, and to study the relationship between these parameters. We show that the *ZNF804A* schizophrenia risk allele is associated with decreased intrahippocampal theta, but increased hippocampal‐PFC coactivation, compared with the nonrisk allele. Furthermore, we show that these parameters are inversely related, irrespective of *ZNF804A* genotype, consistent with a relationship between hippocampal theta and hippocampus‐PFC coactivity.

We show that the *ZNF804A* risk allele is associated with increased coactivation of the hippocampal network and PFC. Previous studies, using seed‐based analyses, have shown that functional connectivity between these regions differs based on *ZNF804A* genotype [Esslinger et al., 2011; Paulus et al., 2013; Rasetti et al., 2011]. However, the direction of effect (i.e., whether the risk allele is associated with greater, or less, functional connectivity, compared with the nonrisk allele), as well as the statistical robustness of this association, varied between studies. Our findings, therefore, add further support for increased coupling of the hippocampus and PFC associated with the risk allele [Esslinger et al., 2009; Paulus et al., 2013], consistent with the increased PFC‐hippocampal coupling seen in patients with schizophrenia [Rasetti et al., 2011].

Our findings demonstrate for the first time, to our knowledge, that a schizophrenia‐associated variant alters hippocampal oscillatory activity. They provide a potential mechanism by which the *ZNF804A*‐related hippocampal‐PFC changes could be mediated. Specifically, decreases in intrahippocampal theta, mediated by the presence of the risk allele, may drive changes in hippocampal‐PFC co‐ordination. Given that human neuroimaging studies of this type are necessarily correlational in nature, it will be of significant interest to investigate hippocampal theta, and its relationship with PFC function, in animal models with altered *ZNF804A* function to establish a direct causal relationship and investigate the underlying cellular and circuitry changes.

It is notable that we found *ZNF804A*‐related changes in hippocampus‐PFC coactivation in the resting state. This is in contrast to a previous study, which showed no *ZNF804A*‐related differences in dorsolateral PFC‐hippocampus functional connectivity at rest [Esslinger et al., 2011]. One reason for this discrepancy may be differences in the analysis approach used. Esslinger et al. [2011] used a seed‐based approach, which examined functional connectivity with a seed region and compared this measure between groups. They showed that *ZNF804A* genotype influenced dorsolateral PFC‐hippocampal functional connectivity during working memory but not during emotional processing or at rest. However, the dorsolateral PFC seed used in these analyses was explicitly defined on the basis of its centering on the voxel showing maximal activity during the working memory task. In contrast, our ICA‐based approach does not require the a priori identification of seed voxels of interest. Instead, dual regression identifies subject‐specific networks derived from an a priori specified set of the well‐established RSNs [Smith et al., 2009]. We then focus on the network of interest (in this case the hippocampal network) and use voxel‐wise tests to compare the extent of each voxel's involvement in the network between genotype groups. Notably, while some regions within the dorsolateral PFC were significantly coactivated with the hippocampal network in our whole group (Fig. 2), the seed voxels (at least the mean seed locations) used by Esslinger et al. were not. Thus, the network based analysis that the ICA‐based approach provides here may be of significant utility for examining the impact of *ZNF804A* (and other variables) under different conditions, as different brain states (e.g., task vs. rest; emotional processing vs. working memory performance) are accompanied by different patterns of network activity. Our data, therefore, suggest that *ZNF804A* modulates hippocampal‐PFC coactivity but that the precise regions of PFC involved may depend on the task (or lack thereof) used. As our data were obtained in resting state, it will be of interest to use ICA‐based analyses to investigate the impact of *ZNF804A* genotype on hippocampal‐PFC coactivity during the performance of cognitive tasks.

It is unclear how *ZNF804A*'s association with schizophrenia [Schizophrenia Working Group of the Psychiatric Genetics Consortium, 2014; Williams et al., 2011] is mediated, as the function of the *ZNF804A* protein is unknown. However, the risk polymorphism affects *ZNF804A* expression during fetal brain development [Hill and Bray, 2012]. *ZNF804A* expression differences during this critical neurodevelopmental window might influence hippocampal circuitry development and ultimately impact on hippocampal theta, as measured here. Hippocampal theta deficits are predicted to impair the temporal co‐ordination of local neuronal assemblies including the cortical gamma rhythms that are also implicated in cognitive processes and dysfunctional in schizophrenia [Uhlhaas and Singer, 2010] resulting in temporally disordered information processing and ultimately in the disturbances of thought and perception seen in patients [Lisman and Buzsaki, 2008]. Consistent with this hypothesis, decreases in hippocampal theta have been observed at the onset of auditory hallucinations in psychosis patients [van Lutterveld et al., 2012]. Although our data were obtained in healthy volunteers, *ZNF804A*‐related differences in hippocampal theta might become pathophysiological in the presence of other genetic and environmental insults, contributing to psychosis.

Our demonstration that intrahippocampal theta and hippocampus‐PFC coactivity are inversely correlated is consistent with the key role that hippocampal theta is proposed to play in co‐ordinating the activity of the hippocampus and PFC [Colgin, 2011; Fujisawa and Buzsaki, 2011]. The correlation was present across all participants (and in each genotype group separately). Any interpretation of this inverse correlation is necessarily speculative; however, it is plausible that it might result from shifts in the patterns of connectivity of the hippocampal network accompanying changes in network states. Thus, increased coupling of hippocampal and PFC activity (such as that which occurs during the performance of cognitive tasks [Benchenane et al., 2010, for example]) might be accompanied by a relative disengagement of hippocampus‐hippocampal network coactivity in the theta band. Shifts in network connectivity patterns are known to occur between different brain states, for example, intrahippocampal theta decreases in active exploration versus sleep [Montgomery et al., 2008]. Therefore, the inverse correlation that we observe may reflect the differential involvement of intrahippocampal versus hippocampus‐PFC connections in different brain states. Furthermore, given that, as discussed above, the precise PFC regions involved likely differ between tasks, it is possible that hippocampal theta‐band activity provides a long‐range signal against which the activity of different PFC regions can be activated or suppressed as appropriate, allowing cortical activity to be precisely tuned and localized according to task demands. Abnormalities in this tuning process could result in the aberrant hippocampal‐PFC connectivity seen in patients with schizophrenia and their relatives [Rasetti et al., 2011].

## CONCLUSIONS

Taken together, our findings demonstrate a decrease in intrahippocampal theta coactivation associated with a *ZNF804A* schizophrenia risk allele and are consistent with this alteration lying upstream of the previously observed changes in hippocampal‐PFC functional connectivity using fMRI. The presence of a correlation between intrahippocampal theta and hippocampal‐PFC coactivation is consistent with the proposed role for hippocampal theta in co‐ordinating activity between the hippocampus and the neocortex. It will be of interest to investigate hippocampal theta, and its phase coupling to PFC neuronal firing, in animal models with disrupted *ZNF804A* function to provide insight into the mechanisms underlying these changes.

## Supporting information

Supplementary Information

Supplementary Information

Supplementary Information
