# Supplementary figures and images for: Modulation of hippocampal theta and hippocampal‐prefrontal cortex function by a schizophrenia risk gene
Source: Hum Brain Mapp. 2015 Mar 10;36(6):2387–95. doi: 10.1002/hbm.22778 (PMC4672713; doi:10.1002/hbm.22778)

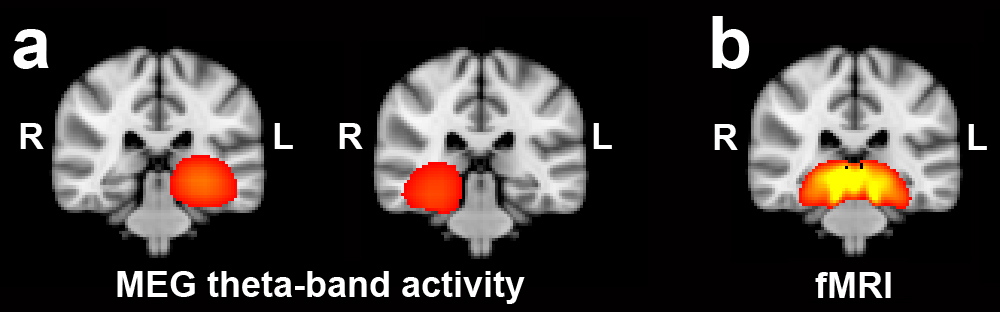

Supplement: Supplementary file 1 — Supplementary Information [file HBM-36-2387-s001.tif]

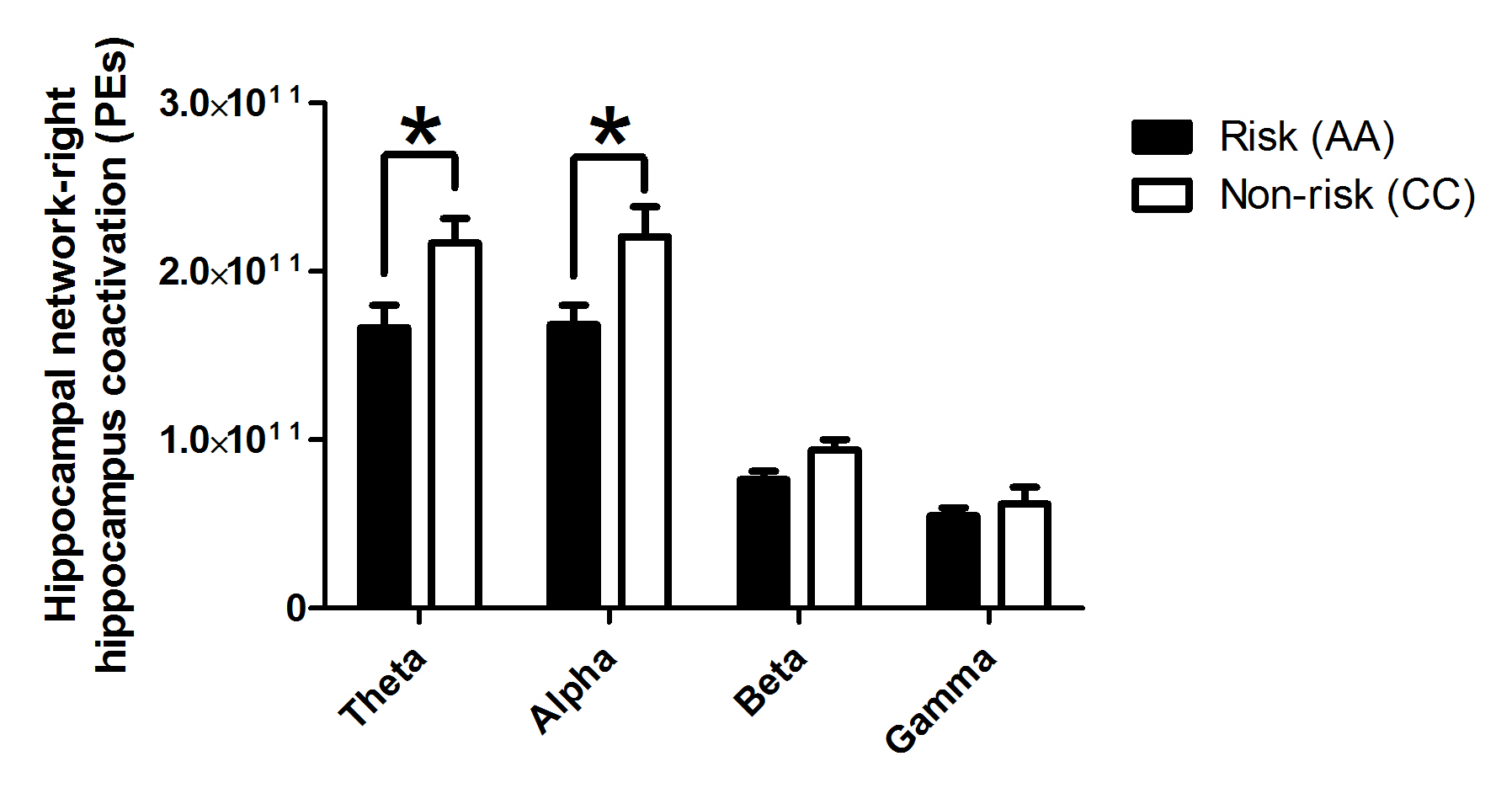

Supplement: Supplementary file 2 — Supplementary Information [file HBM-36-2387-s002.tif]

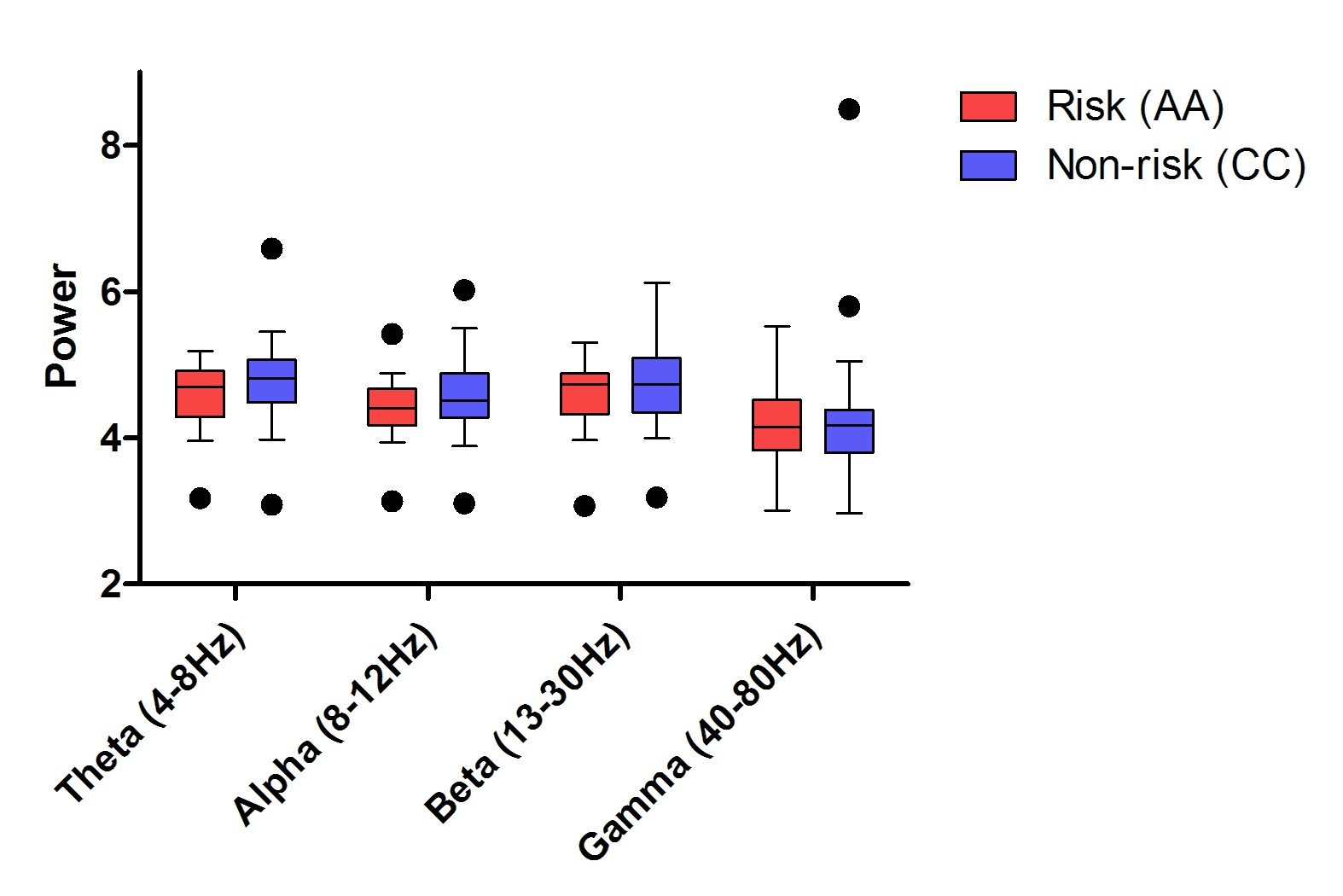

Supplement: Supplementary file 3 — Supplementary Information [file HBM-36-2387-s003.tif]
